# Neuropeptide S Counteracts Paradoxical Sleep Deprivation-Induced Anxiety-Like Behavior and Sleep Disturbances

**DOI:** 10.3389/fncel.2018.00064

**Published:** 2018-03-06

**Authors:** Jun-Fan Xie, Yu-Feng Shao, Hai-Liang Wang, Can Wang, Guang-Fu Cui, Xiang-Pan Kong, Lin-Xin Wang, Yu-Nong Chen, Chao-Yu Cong, Hai-Lin Chen, Yi-Ping Hou

**Affiliations:** ^1^Departments of Neuroscience, Anatomy, Histology, and Embryology, Key Laboratory of Preclinical Study for New Drugs of Gansu Province, School of Basic Medical Sciences, Lanzhou University, Lanzhou, China; ^2^Department of Human Anatomy, School of Medicine, Hunan Normal University, Changsha, China

**Keywords:** neuropeptide S, neuropeptide S receptor, paradoxical sleep deprivation, anxiety-like behavior, sleep-wake states, amygdala

## Abstract

Disturbed sleep is a common subjective complaint among individuals with anxiety disorders. Sleep deprivation increases general and specific anxiety symptoms among healthy individuals. The amygdala is critical for regulating anxiety and also involved in mediating the effects of emotions on sleep. Neuropeptide S (NPS) and NPS receptors (NPSR) are reported as a novel endogenous arousal and anxiolytic system, but it is unclear yet whether this system is involved in anxiety-like behavior and sleep caused by sleep deprivation, and how it plays anxiolytic effect underlying the comorbid condition. In the present study, we demonstrate that paradoxical sleep deprivation (PSD) induced by modified multiple platform method (MMPM) for 24 h caused anxiety-like behavior, a prolonged sleep latency and subsequent paradoxical sleep (PS) rebound accompanied by an increase in electroencephalogram (EEG) theta (4.5–8.5 Hz) activities across light and dark phase in rats. The increase of PS after PSD was due to an increase of episode number during light phase and both episode number and duration during dark phase. Central action of NPS (1 nmol) attenuated PSD-induced anxiety-like behavior, and altered PSD-induced sleep-wake disturbances through increasing wakefulness, and suppressing PS and EEG theta activities. The reduction in PS time following NPS administration during light phase was because of a decreased episode number. Furthermore, sleep amount in 24 h in PSD rats given NPS was lesser than that given saline. PSD significantly enhanced NPSR mRNA expression level in the amygdala. NPS remarkably increased the number of Fos-ir neurons in the basolateral amygdala (BLA), the central amygdala (CeA) and medial amygdala (MeA). The majority of Fos-ir neurons induced by NPS also expressed NPSR. These results suggest that NPSR upregulation in the amygdala is presumably related to the PSD-induced anxiety-like behavior and sleep disturbances, and that NPS counteracts PSD-induced anxiety-like behavior and sleep disturbances possibly through activating the neurons bearing NPSR in the amygdala. In addition, the little sleep increase in PSD rats treated with NPS suggests that NPS can function as an anxiolytic without causing a subsequent sleep rebound.

## Introduction

Neuropeptide S (NPS), a 20-amino acid neuropeptide highly conserved among mammals, is mainly produced in a group of neurons located in the peri-locus coeruleus of the brainstem and exerts its effect in the brain regions where NPS receptor (NPSR) is expressed (Xu et al., 2004). The profile of NPSR expression suggests the involvement of NPS-NPSR system in the regulation of multiple central functions. Activation of NPSR by NPS promotes wakefulness (Xu et al., 2004; Zhao et al., 2012) and evokes anxiolytic-like effects in rodent (Xu et al., 2004; Duangdao et al., 2009; Enquist et al., 2012). NPS is also involved in fear expression and extinction (Jüngling et al., 2008; Fendt et al., 2010), antinociception (Zhang et al., 2014), and facilitation of olfactory function (Shao et al., 2013) and memory (Shao et al., 2016).

Disturbed sleep is a common subjective complaint among individuals with anxiety disorders (Monti and Monti, 2000; Maclean and Datta, 2007). In patients with psychiatric disorders, most notably anxiety, insomnia is the most commonly reported sleep disturbance (Szelenberger and Soldatos, 2005). Sleep deprivation causes general and specific anxiety symptoms in healthy individuals (Sagaspe et al., 2006; Babson et al., 2010), and 70% of the people suffering from anxiety disorders are also sleep deprived (Wyatt et al., 1971). Several animal studies suggest that paradoxical sleep deprivation (PSD), also known as rapid eye movement sleep deprivation (REMSD; Maclean and Datta, 2007; Vollert et al., 2011; Pires et al., 2013), and total sleep deprivation (Xu et al., 2010; Pires et al., 2013) cause anxiety-like behavior.

Evidence from human studies suggests that the amygdala plays an important role in anxiety. The basolateral amygdala (BLA) and the central amygdala (CeA) are especially involved in anxiety and fear (Grupe and Nitschke, 2013; Tovote et al., 2015). Sleep deprivation-induced sleepiness enhanced amygdala response to subliminal signals of anxiety and fear (Motomura et al., 2014). The amygdala, compared with other brain regions, has higher expression levels of NPSR and is the main site of action of the anxiolytic effect of NPS (Xu et al., 2004; Slattery et al., 2015; Zhang et al., 2016; Zoicas et al., 2016). Moreover, the amygdala is also involved in the regulation of sleep and arousal, especially in the regulation of stress-induced alterations in sleep (Wellman et al., 2014).

The present experiments were designed to investigate the effects of intracerebroventricular (i.c.v.) administration of NPS on PSD-induced anxiety-like behavior and sleep-wake profile. The alterations of NPSR mRNA expression induced by PSD and the activation of neurons induced by NPS in the amygdala were analyzed using real-time qPCR and c-Fos *ex-vivo* immunohistochemistry (IHC) for revealing the potential mechanism of NPS-NPSR system involved in PSD-induced anxiety-like behavior and the effect of NPS on it. The dual-immunofluorescence staining of c-Fos and NPSR was performed to determine whether the neurons activated by NPS are ones that also express NPSR.

## Materials and Methods

### Animals

Male Sprague-Dawley rats, weighing 250–300 g (8–10 weeks old), were purchased from the Experimental Animal Center of Lanzhou University (Lanzhou, China). Upon arrival at the animal housing facility, they were housed in groups of four in plastic cages (485 mm L × 350 mm W × 225 mm H) and kept in an automatically controlled room on a 12:12-h light/dark cycle (lights on 8:00–20:00 h, illumination intensity = 100 lx) at an ambient temperature (22 ± 1°C) and 50% relative humidity with food and water available *ad libitum*. All animals were cared for, and experiments were conducted in accordance with the National Institutes of Health Guide for the Care and Use of Laboratory Animals (1996 revision). The experimental protocol was approved by the Ethics Committee of Lanzhou University (permit number: SCXK Gan 2013-0002). All possible efforts were made to reduce the number of animals used and discomfort to the animals. Rats were acclimatized for 5 days before any experiment. Only once did the rats undergo PSD and receive injection.

### Experimental Design and Procedure

**Figure 1:**
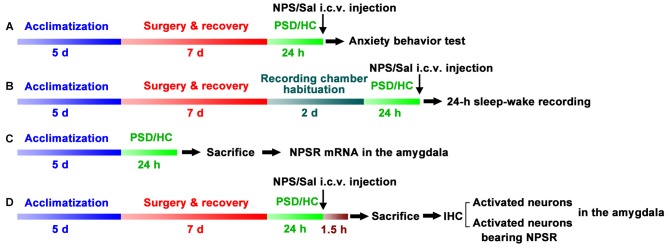
Schematic representation of the experimental design and procedures. Anxiety-like behavior tests **(A)**, 24-h sleep-wake recording **(B)**, quantifying Neuropeptide S (NPS) receptor (NPSR) mRNA in the amygdala **(C)** and immunohistochemistry (IHC) of c-Fos and NPSR to detect activated neurons and determine whether activated neurons bear NPSR **(D)** in the amygdala were applied to investigate paradoxical sleep deprivation (PSD)-induced anxiety-like behaviors and the effect of centrally administered NPS on them. Rats in experiments **(A–D)** except home cage control (HC) ones were subjected to PSD for 24 h using the modified multiple platform method (MMPM). NPS or saline (Sal) i.c.v. administration was carried out 5 min before anxiety-behavior tests **(A)** and sleep-wake recording **(B)**. In experiment **(C)**, bilateral amygdala was harvested immediately after the rats were sacrificed to detect NPSR mRNA using RT-qPCR. Animals in experiment **(D)** were sacrificed following NPS or Sal i.c.v. administration, and their brains were sectioned and immunostaining was performed to detect NPS activated neurons in the amygdala and determine whether these neurons express NPSR.

Four experiments were conducted as follows: (A) Anxiety-like behavior were examined using open field test (OFT) and light-dark box (LDB) test in home cage control (HC) rats treated with NPS and saline, as well as PSD rats treated with NPS and saline (*n* = 9–12 in each group, Figure 1A). (B) Sleep-wake cycle was recorded in HC rats treated with saline (*n* = 8), as well as PSD rats treated with NPS (*n* = 8) and saline (*n* = 9, Figure 1B). (C) Rats NPSR mRNA levels in the amygdala were determined in HC (*n* = 5) and PSD rats (*n* = 5, Figure 1C). (D) IHC for c-Fos to reveal NPS-induced activated neurons, and dual-immunofluorescence staining for c-Fos and NPSR to reveal corelationship of activated neurons and NPSR expression in the amygdala were performed in HC and PSD rats treated with NPS (*n* = 5) and saline (*n* = 5, Figure 1D).

### Surgeries

For rats used for sleep-wake cycle recording, under chloral hydrate anesthesia (350 mg/kg, i.p.), four stainless steel screw cortical electrodes (1 mm diameter) were screwed through the skull into frontal (2 mm lateral and anterior to the bregma) and parietal (2 mm lateral to the lambda) cortices to record electroencephalogram (EEG). Three silver wires were inserted into the dorsal cervical neck muscles to record electromyogram (EMG). The free ends of the electrode leads and silver wires were soldered into a 7-pin miniature plug. For i.c.v. injection, a guide cannula (0.6 mm diameter, 20 mm long) was stereotaxically implanted into the right lateral ventricle (AP −0.9, ML +1.5, DV −3.3) of the rat according to the atlases of Paxinos and Watson (1998). The plug and cannula were chronically fixed to skull with dental cement. After surgeries, animals were allowed to recover for 1 week.

### Drug Administration

NPS (rat, SFRNGVGSGVKKTSFRRAKQ) was synthesized by Shanghai Mocell Biotech Co. Ltd. Shanghai, China and freshly dissolved in saline before use. The dose of NPS (1 nmol in 5 μl of saline per animal) was chosen based on our previous study in which this dose promoted wakefulness in rats (Zhao et al., 2012). NPS or equal volume of saline was administrated into the lateral ventricle through the planted guide cannula at the speed of 1 μl/min 5 min prior to the behavior test or sleep-wake recording at 8:00 h (Zhao et al., 2012; Shao et al., 2013, 2016).

After the experiments, 1 μl of methylene blue dye was injected into ventricle via guide cannula 5 min before the rats were decapitated under deep anesthesia with chloral hydrate. Brains were removed and frozen. Gross dissection of the brain was used to verify the site of NPS and saline administration. Only the data from animals with dye dispersion throughout the ventricle were used.

### Paradoxical Sleep Deprivation (PSD)

The PSD procedure was conducted with the modified multiple platform method (MMPM) as described in previous studies (Machado et al., 2004; Silva et al., 2004; Vollert et al., 2011; Pires et al., 2013). Briefly, in order to get 24-h PSD, four rats were placed inside a tiled water tank (120 cm L × 44 cm W × 44 cm H) containing 15 platforms, 5 cm in diameter, spaced 7 cm apart, and surrounded by water up to 2 cm beneath the surface. Food and water were available *ad libitum* through a grid placed on top of the water tank. The animals were group-housed which allows them to form stable social groups and reduces chances of psychosocial or isolation stress within the tank environment (Vollert et al., 2011). Rats were capable of moving inside the tank and jumping from one platform to another. When a rat enters the paradoxical sleep (PS), it falls into the water due to muscle atonia and wakes up. It has been previously demonstrated that this protocol suppresses 96%–100% of PS in rodent (Machado et al., 2004; Silva et al., 2004). Home cage (HC) animals (four rats per cage) were maintained in their cages in the same room.

### Tests of Anxiety-Like Behavior

After PSD, the rats were taken out of water tank, gently dried with a soft towel, and promptly moved to the area of the room where anxiety test equipment was located 30 min before the tests. There, they were left to air dry for 25 min and kept awake during this period. The rats were then treated with NPS or saline before the anxiety-like behavior was evaluated with either OFT or LDB test. Before each behavior test, the apparatus was cleaned with 70% ethanol, wiped with hand towels to eliminate possible odors left by previous rats.

The OFT was conducted in the 24-h PSD rats treated with NPS or saline, as well as HC rats treated with NPS or saline. Each rat was placed in the center of a 60 cm × 60 cm open field surrounded by 40 cm high wall in standard room lighting conditions (illumination, 120 lux) and was left free to explore the arena for 5 min. Activities were recorded using computer-operated motion tracking system R. D. BehaviorSys v2.8.7 (Mobiledatum, Shanghai, China) that utilizes a digital video camera to track and score the animal behaviors. The program tabulated center time, the periphery time, distance traveled, grooming and rearing. The center time was the time the rat spent in the center zone which is 25 cm × 25 cm square in the center of the open-field arena. Grooming is a behavior described as a rat washing the face or any other part of its body with the forepaw, and rearing is defined as a rat standing on its hind legs (Andersen et al., 2005).

The LDB used for the test consists of a light compartment (27 cm W × 27 cm L × 27 cm H, illumination 100 lux) and a dark compartment (black colored surrounding walls and floor, 27 cm W × 18 cm L × 27 cm H, illumination 2 lux) separated by a partition with a single opening (7 cm × 7 cm) for passage from one compartment to the other. A rat was placed in the light compartment of LDB, and was monitored for 5 min to measure the number of transitions and total time spent in the light compartment with R. D. BehaviorSys. A transition is defined as entering the light or dark box when both front paws and shoulders are inside the respective compartment. Rats are nocturnal and prefer darker areas, and a decrease in the exploratory activity in a lighted area is believed to be indicative of increased anxiety-like behavior and the time spent in the light is considered as a measure of anxiety-like behavior (Vollert et al., 2011).

### Polygraphic Recordings and Sleep-Wake States Analysis

One week after surgery, the rats for sleep-wake cycle recording were placed in a sound-attenuated, ventilated and electrically isolated chamber, and were connected to recording cable attached to a slip ring for a habituation period of 2 days. The rats were then moved out of the chamber to undergo PSD with MMPM or kept in their home cage in the same room for 24 h. After that, the PSD and control rats were transferred from water tank or home cage to the recording chamber, and again, connected to the recording cable 30 min before 24-h sleep-wake recording (8:00 h–8:00 h).

EEG and EMG activities were amplified (2000×) and filtered (0.5–60 Hz for EEG and 30–300 Hz for EMG, Model 3500, A-M Systems, WA, USA), and digitalized at a resolution of 256 and 128 Hz and recorded continuously with CED 1401 MK II (Cambridge Electronic Design Limited (CED), London, UK). The behaviors of the rats during the light and dark phases in the chamber were monitored and recorded by an infrared video camera. Using a Spike 2 sleepscore script (CED) and with the assistance of spectral analysis by the fast Fourier transform (FFT), we visually scored polygraphic records by 30-s epochs for wakefulness (W), slow wave sleep (SWS) and PS according to our previously described criteria validated for rats (Zhao et al., 2012).

### Real-Time Quantitative PCR (RT-qPCR)

The PSD rats or home cage control rats under deep anesthesia were decapitated on the ice. Brain was quickly removed and frozen in liquid nitrogen. Coronal sections of 300 μm thickness were cut from frozen brain on a cryostat. The bilateral amygdalae were punched out using a stainless steel punch needle (1.6 mm in diameter) from sections between −1.6 mm and −3.6 mm relative to the bregma based on the coordinates of rat atlases of Paxinos and Watson (1998), and the tissue was harvested and stored at −80°C until RNA isolation. These procedures were performed under RNase-free conditions. The rest of the brain sections after the amygdala punched was fixed with 4% paraformaldehyde (PFA) in 0.1 M phosphate buffer (PB, pH 7.4) for 24 h, and subsequently immersed in 30% sucrose solution in 0.1 M PB at 4°C for 48 h and coronally sectioned (30 μm) on a cryostat (Thermo Fisher, USA) at −20°C. These sections were stained with cresyl violet to identify the scope of tissues punched (Figures 6A–C). In 10 rats, the punched tissue samples only involved in the amygdala were used for RT-qPCR to detect NPSR mRNA. In two rats, tissue samples included regions other than amygdala were not used for RT-qPCR analyses.

**Figure 2:**
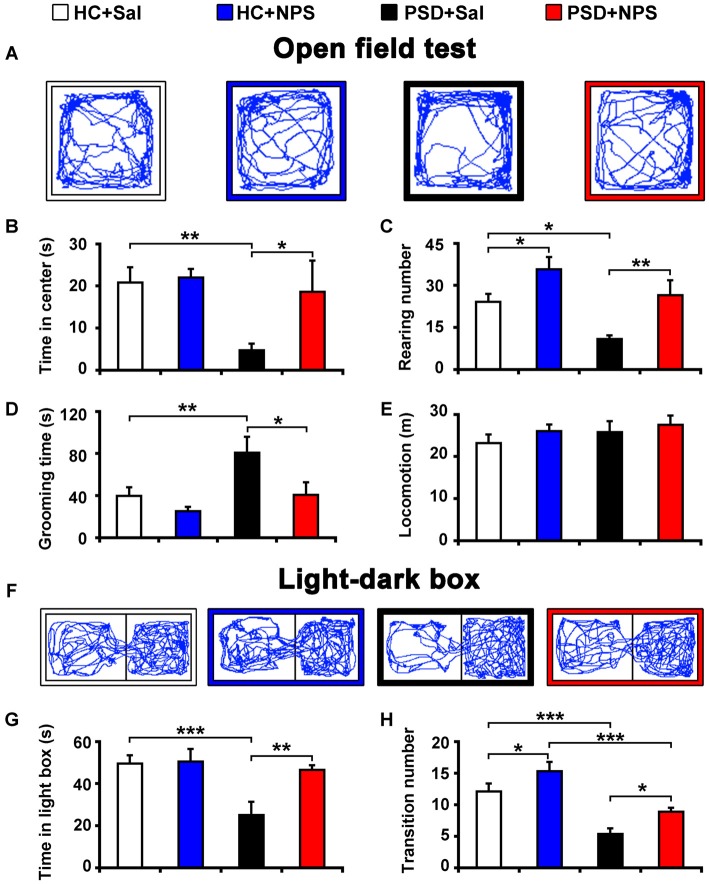
Central administration of NPS attenuated PSD-induced anxiety-like behaviors. After 24-h PSD, rats were subjected to open field test (OFT) **(A–E)** and light-dark box (LDB) **(F–H)** anxiety tests. NPS or saline (Sal) i.c.v. injection was carried out 5 min before tests. Representative locomotor paths in OFT **(A)** and LDB **(F)** of home cage control rats given saline (HC + Sal) or NPS (HC + NPS), and PSD rats given saline (PSD + Sal) or NPS (PSD + NPS) are respectively shown. PSD decreased the time spent in center zone **(B)** and rearing number **(C)**, but increased the time spent in grooming **(D)** in OFT. PSD also decreased the time spent in light box **(G)** and transition number **(F,H)** in LDB. NPS abolished all the changes caused by PSD **(A–D,F–H)**. There was no significant difference in locomotion in the OFT among four groups **(E)**. Values are means ± SEM (*n* = 9–12). *P < 0.05, **P < 0.01, ***P < 0.001. Data were analyzed by two-way analyses of variance (ANOVA).

**Figure 3:**
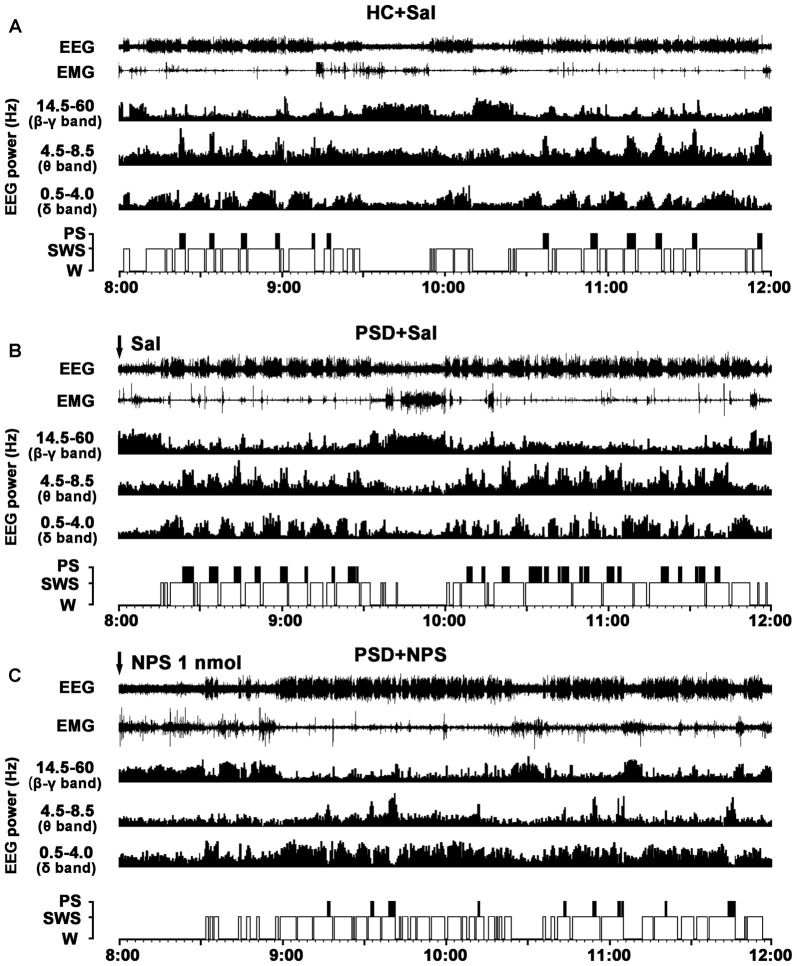
Effects of NPS i.c.v. injection on the EEG and sleep-wake states after 24-h PSD. Representative 4-h (8:00–12:00) EEG, electromyogram (EMG), hypnograms, as well as cortical power spectra at 0.5–4, 4.5–8.5 and 14.5–60 Hz in HC + Sal **(A)**, PSD + Sal **(B)** and PSD + NPS **(C)** are shown. Note that the PSD + Sal rat had a long sleep latency characterized by fast and low-voltage activities in cortical EEG, as well as dense activity in EMG at the first hour, and subsequent marked paradoxical sleep (PS) and slow wave sleep (SWS) recovery as compared to HC + Sal rat. The PSD + NPS rat displayed an increase in wakefulness accompanied with increased cortical 14.5–60 Hz activities and suppressed cortical 4.5–8.5 and 0.5–4.0 Hz activities for about 30 min. After that, there was a SWS recovery accompanied with increased 0.5–4.0 Hz activities of cortical EEG.

**Figure 4:**
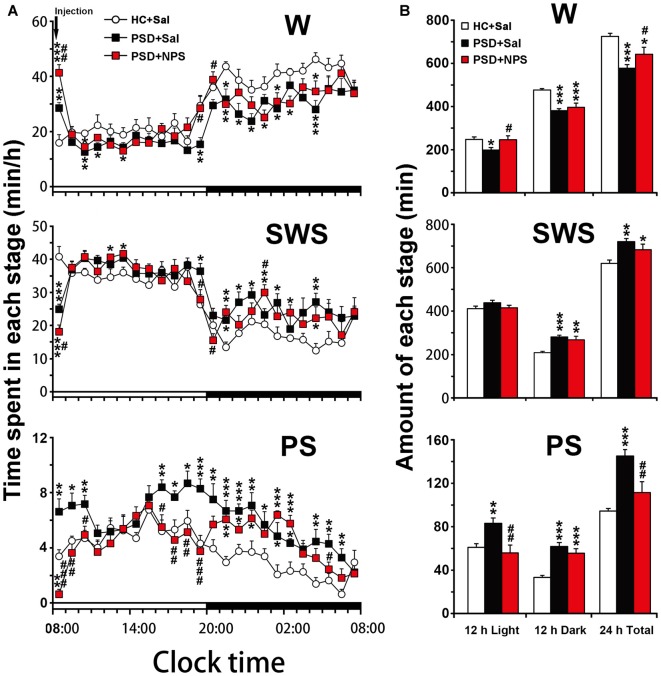
Sleep-wake profiles of home cage control (HC) rats given saline (Sal), as well as PSD rats treated with Sal and NPS. Time-course changes in wakefulness (W), SWS and PS for 24 h **(A)**. The horizontal open and filled bars indicate the 12 h light and 12 h dark periods, respectively. Total time spent in W, SWS and PS during the 12 h light, 12 h dark phases and 24 h total **(B)**. Data are the means ± SEM (*n* = 8–9). *P < 0.05, **P < 0.01, ***P < 0.001 compared to HC + Sal; ^#^*P* < 0.05, ^##^*P* < 0.01, ^###^*P* < 0.001, compared to PSD + Sal. Statistics were analyzed by one-way ANOVA and followed by Fisher’s LSD test.

**Figure 5:**
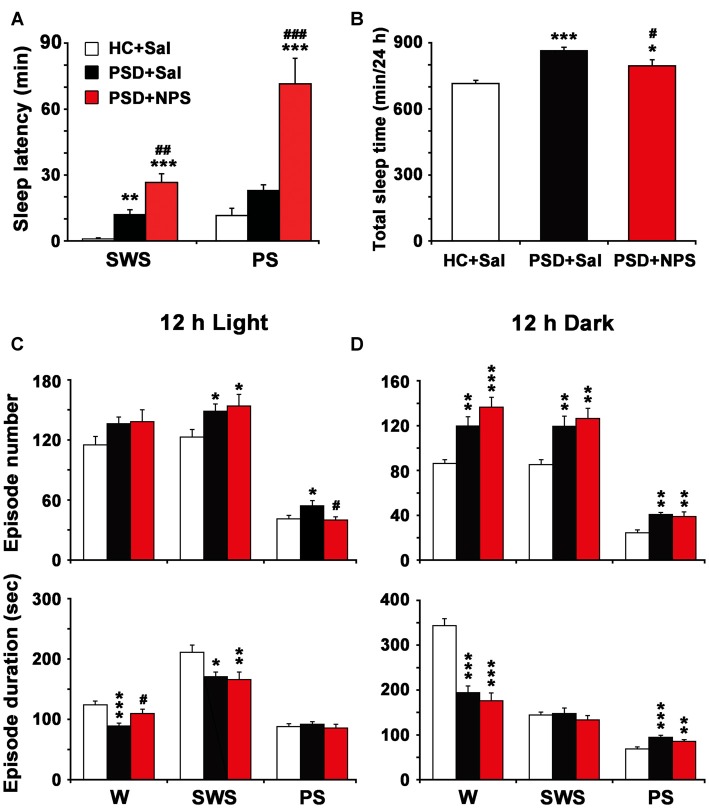
Sleep latency **(A)**, total sleep time in 24 h **(B)** and episode number and duration of each stage during 12 h light **(C)** and 12 h dark periods **(D)**. Values are the means ± SEM (*n* = 8–9). *P < 0.05, **P < 0.01, ***P < 0.001 compared to HC + Sal; ^#^*P* < 0.05, ^##^*P* < 0.01, ^###^*P* < 0.01, compared to PSD + Sal. Statistics were analyzed by one-way ANOVA and followed by Fisher’s LSD test.

**Figure 6:**
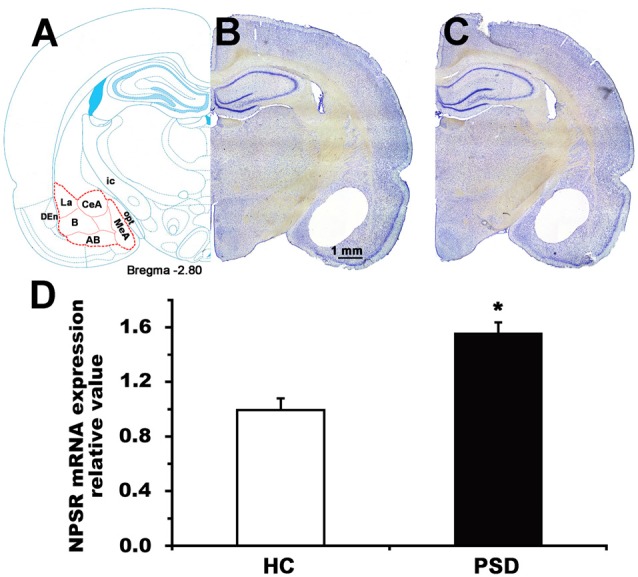
NPSR mRNA expression level of the amygdala in control and 24-h PSD rats. Schematic representation **(A)** shows the area (red dashed line) sampled for mRNA measurement in the coronal section at −2.80 mm from bregma (Paxinos and Watson, 1998). Representative photographs **(B,C)** show blank area from where the samples of amygdala were punched in home cage control (HC) and PSD rats, respectively. Histograms **(D)** shows presented the relative value of NPSR mRNA expression in HC and PSD rats. Values are means ± SEM (*n* = 5 per group). *P < 0.01. Data were analyzed by Student’s *t*-test. Abbreviations: AB, accessory basal nuclei; B, basal nuclei; CeA, central amygdala; DEn, dorsal endopiriform nuclei; ic, internal capsule; La, lateral nuclei; MeA, medial amygdala; opt, optic tract.

Total RNA was isolated from the amygdaloid tissue using Trizol reagent (Invitrogen, USA) according to the manufacturer’s instruction, and was quantified by spectrophotometric absorption at 260 nm (Eppendorf Biophotometer, Germany). The cDNA was synthesized from 500 ng of total RNA using PrimeScript™ RT Master Mix (RR036A, Takara, Japan).

RT-qPCR was performed using SYBR premix EX Tag™ II (RR820A, Takara, Japan) on Thermo PikoReal 96 Real-Time PCR System (Thermo Scientific, USA) under the following cycle steps: initial denaturation at 95°C 2 min, and 40 cycles of denaturation at 95°C 5 s and annealing/extension at 60°C 1 min. The PCR primers were designed based on gene sequences from PubMed: rat NPSR (fwd: 5′-TGCAGAATCGTCCGCTACTTACA-3′, rev: 5′-TCCGATGAGGACTTTGGCTTG-3′); Glyceraldehyde-3-phosphate dehydrogenase (GAPDH) was used as internal control (fwd: 5′-GGCACAGTCAAGGCTGAGAATG-3′, rev: 5′-ATGGTGGTGAAGACGCCAGTA-3′). The qPCR was performed in triplicate. The RT-qPCR data were analyzed using Thermo PikoReal Software. The 2^−ΔΔCt^ method was utilized to determine relative amount of mRNA, and the result from each sample was normalized to the housekeeping gene GAPDH and expressed as ratio to that of control group.

### Immunohistochemistry

#### Tissue Preparation

Ninety minutes after the NPS (1 nmol, *n* = 5) or saline (*n* = 5) i.c.v. administration, the HC and PSD rats were anesthetized with over dose chloral hydrate (400 mg/Kg) and perfused via the ascending aorta with 200 ml saline followed by 300 ml ice-cold 4% PFA in 0.1 M PB (pH 7.4). The brains were removed, post-fixed in 4% PFA overnight, and immersed in 30% sucrose solution in 0.1 M PB at 4°C for 48 h, and then coronally sectioned (30 μm) on a cryostat (Thermo Scientific, USA) at −20°C.

#### Fos Immunostaining for Revealing NPS-Induced Neuronal Activation

The floating sections were rinsed in 0.01 M PB saline (PBS, pH 7.4), processed 30 min in 0.3% H_2_O_2_ in PBS, and incubated in blocking buffer (10% bovine serum in PBS) for 1 h. Then the sections were incubated with rabbit polyclonal antibody against c-Fos (1:6000, sc-253, Santa Cruz Biotechnology, Santa Cruz, USA) in PBS containing 1% bovine serum for 72 h at 4°C on an agitator. After rinsing in PBS, sections were incubated with a biotinylated goat anti-rabbit IgG (1:1000, AP132B, Millipore, Temecula, CA, USA) for 48 h at 4°C and then with horseradish peroxidase conjugated streptavidin (1:2000, SA202, Millipore, Temecula, CA, USA) for 24 h at 4°C. Following rinsing, the sections were immersed in 0.05 M Tris-HCl buffer (pH 7.6), containing 0.05% 3, 3′ diaminobenzidine (DAB), 0.01% H_2_O_2_, and 0.6% nickel ammonium sulfate for 2–5 min at room temperature. The sections were mounted on gelatin-coated glass slides, dried, dehydrated and covered with a coverslip, using DPX, for light microscopy.

#### Dual-Immunofluorescence for Co-Expression of Fos and NPSR in the Amygdala

These sections were blocked with 10% bovine serum in PBS before they were incubated with a mixture of rabbit polyclonal antibody against c-Fos (1:1500) and goat anti-NPSR (1:500, sc-162893, Santa Cruz Biotechnology, Santa Cruz, CA, USA) in PBS containing 1% bovine serum for 72 h at 4°C on an agitator. After several rinses in PBS, the sections were incubated with Alexa Fluor^®^ 488-conjugated affinipure donkey anti-rabbit IgG (1:400, 711–545–152, Jackson ImmunoResearch Laboratories, Inc., PA, USA) and Cy™ 3-conjugated affinipure donkey anti-goat IgG (1:400, 705–165–147, Jackson ImmunoResearch Laboratories, Inc., PA, USA) for 24 h at 4°C. Primary antibody omission was used as a control. Finally, sections were mounted on slides, covered with a coverslip, using 50% glycerol in PBS, and observed under a fluorescence microscope and photographed under Zeiss LSM 710 laser confocal microscope. The specificity of the anti-NPSR antibody had been demonstrated in previous studies (Laitinen et al., 2004; Shao et al., 2013, 2016).

### Data Analysis

#### Cell Counting

The BLA, CeA and medial amygdala (MeA) within sections at bregma −1.80, −2.30, −2.80, −3.30 mm were determined by the characteristics of their cytoarchitecture and peripheral white matter according to the rat atlases of Paxinos and Watson (1998). In these areas of amygdala of both sides, Fos-ir neurons under light microscope, Fos and NPSR immunofluorescent neurons and co-expression neurons under confocal microscope were counted with counting tool of ZEISS Efficient Navigation (ZEN) microscope software (Germany). The average of numbers from two sides of each rat was calculated and used for analysis.

#### Statistical Analysis

All data were expressed as means ± SEM. Statistical significance was analyzed by two-way analysis of variance (ANOVA) for behavioral test, and the number of Fos-ir neurons and the percentage of co-expression of Fos- and NPSR-ir neurons, one-way ANOVA followed by *post hoc* Fisher’s least significant difference (LSD) test for sleep parameters, and student’s *t* test for RT-qPCR results. The significance was set at *p* < 0.05.

## Results

### Effect of NPS on PSD-Induced Anxiety-Like Behavior

To examine the effects of NPS on anxiety-like behavior, OFT and LDB were used in the present study.

PSD for 24 h increased anxiety as assessed by OFT. Analysis of the data by two-way ANOVA revealed a significant sleep condition effect (*F*_(1,35)_ = 5.456, *p* < 0.05), but no drug effect (*F*_(1,35)_ = 3.254, *p* = 0.08) or sleep condition × drug interaction (*F*_(1,35)_ = 2.297, *p* > 0.05) in the time spent in center zone of open field. Analysis for rearing number and the time spent in grooming respectively indicated a significant sleep condition (*F*_(1,35)_ = 9.590, *p* < 0.01; *F*_(1,35)_ = 6.887, *p* < 0.05) and drug effect (*F*_(1,35)_ = 14.273, *p* < 0.001; *F*_(1,35)_ = 6.483, *p* < 0.05), but no sleep condition × drug interaction (*F*_(1,35)_ = 0.301, *p* > 0.05; *F*_(1,35)_ = 1.380, *p* > 0.05). In comparison with HC rats given saline, PSD rats given saline showed less time in center zone (4.75 ± 1.58 s vs. 20.84 ± 3.59 s; *F*_(1,35)_ = 8.025, *p* < 0.01; Figures 2A,B) and decreased rearing number (10.90 ± 1.35 vs. 24.09 ± 2.84; *F*_(1,35)_ = 7.189, *p* < 0.05; Figure 2C), but an increase in the time of grooming (80.82 ± 15.07 s vs. 39.95 ± 8.00 s; *F*_(1,35)_ = 7.808, *p* < 0.01; Figure 2D). However, there was no significant difference in total distance traveled among four groups (Figures 2A,E). NPS (1 nmol) i.c.v. administration to PSD rats showed anxiolytic effect. Compared to PSD rats given saline, NPS increased the time spent in center zone (18.62 ± 7.43 s vs. 4.75 ± 1.58 s; *F*_(1,35)_ = 5.391, *p* < 0.05; Figures 2A,B) and rearing number (26.56 ± 5.29 vs. 10.9 ± 1.35; *F*_(1,35)_ = 9.175, *p* < 0.01; Figure 2C), but decreased the time spent in grooming (40.79 ± 12.09 s vs. 80.82 ± 15.07 s; *F*_(1,35)_ = 6.774, *p* < 0.05; Figure 2D). Furthermore, analysis between HC rats treated with saline and NPS showed that NPS increased rearing number (35.78 ± 4.36 vs. 24.09 ± 2.84; *F*_(1,35)_ = 5.332, *p* < 0.05; Figure 2C), and did not alter the time spent in center zone and grooming (Figures 2A,B,D). No significant difference in each parameter in OFT was found between HC and PSD rats treated with NPS (Figures 2A–E).

PSD rats also demonstrated anxiety when assessed with LDB test. Analysis of the data by two-way ANOVA revealed a significant sleep condition effect (*F*_(1,39)_ = 9.370, *p* < 0.01), and drug effect (*F*_(1,39)_ = 5.801, *p* < 0.05) and sleep condition × drug interaction (*F*_(1,39)_ = 4.864, *p* < 0.05) in the time spent in light box. Analysis for transition number indicated a significant sleep condition (*F*_(1,39)_ = 36.932, *p* < 0.001) and drug effect (*F*_(1,39)_ = 9.774, *p* < 0.01), no sleep condition × drug interaction (*F*_(1,39)_ = 0.020, *p* > 0.05). PSD rats given saline decreased the time spent in light box (25.03 ± 6.39 s vs. 49.59 ± 3.90 s; *F*_(1,39)_ = 13.594, *p* < 0.001; Figures 2F,G) and transition number (5.40 ± 0.85 vs. 12.09 ± 1.27; *F*_(1,39)_ = 18.961, *p* < 0.001; Figures 2F,H) compared to HC rats given saline. NPS administration reversed the PSD-induced anxiety-like behavior. It increased the time spent in light box (46.55 ± 2.14 s in PSD + NPS vs. 25.03 ± 6.39 s in PSD + Sal; *F*_(1,39)_ = 10.865, *p* < 0.01; Figures 2F,G) and transition number (8.92 ± 0.63 in PSD + NPS vs. 5.40 ± 0.85 in PSD + Sal; *F*_(1,39)_ = 5.454, *p* < 0.05; Figures 2F,H). In addition, analysis between HC rats given saline and NPS showed that NPS increased transition number (15.30 ± 1.45 vs. 12.09 ± 1.27; *F*_(1,39)_ = 4.362, *p* < 0.05; Figures 2F,H), and did not change the time spent in light box (Figures 2F,G). PSD rats given NPS decreased transition number (8.92 ± 0.63 vs. 15.30 ± 1.45; *F*_(1,39)_ = 17.971, *p* < 0.001; Figures 2F,H), and did not influence the time spent in light box as compared to HC rats given NPS (Figures 2F,G).

### Effect of NPS on PSD-Induced Sleep-Wake Architecture

#### The Characteristic of PSD-Induced Sleep-Wake Pattern

In comparison with HC rats given saline (Figure 3A), the PSD rats treated with saline (Figure 3B) had a longer sleep latency accompanied by fast and low-voltage activity in cortical EEG and a dense EMG activity, followed by an increase in PS during sleep recovery. Power spectral analysis of cortical EEG during sleep recovery showed that PSD promoted theta (4.5–8.5 Hz) and delta (0.5–4 Hz) activities, and reduced beta and gamma (14.5–60 Hz) activities.

An analysis of hourly amount of time spent in each stage over 24 h revealed that PSD decreased wakefulness during both light and dark period except the first hour compared to HC rats given saline. Meanwhile, it increased SWS mainly during dark period and PS during 8:00–10:00 h and 14:00–19:00 h of clock time of light period and across dark period (Figure 4A). An analysis of amount of each stage in 12-h light and dark phase as well as 24-h total showed that PSD increased PS (Figure 4B lower) by 37% in 12-h light phase (*F*_(2,22)_ = 7.803, *p* = 0.003; post-test, *p* < 0.01), 85% in 12-h dark phase (*F*_(2,22)_ = 19.174, *p* < 0.001; post-test, *p* < 0.001), and 54% in 24-h total (*F*_(2,22)_ = 14.674, *p* < 0.001; post-test, *p* < 0.001). PSD increased SWS (Figure 4B middle) by 34% during 12-h dark period (*F*_(2,22)_ = 12.247, *p* < 0.001; post-test, *p* < 0.001) and 16% for 24-h total (*F*_(2,22)_ = 7.418, *p* = 0.003; post-test, *p* < 0.01), respectively. Meanwhile, wakefulness (Figure 4B upper) was decreased by 20% in 12-h light phase (*F*_(2,22)_ = 4.512, *p* = 0.023; post-test, *p* < 0.05), 20% in 12-h dark phase (*F*_(2,22)_ = 16.583, *p* < 0.001; post-test, *p* < 0.001) and 20% in 24-h total (*F*_(2,22)_ = 11.303, *p* < 0.001; post-test, *p* < 0.001).

As shown in Figure 5A, PSD rats given saline increased SWS latency (11.94 ± 2.25 min vs. 0.94 ± 0.41 min; *F*_(2,22)_ = 23.069, *p* < 0.001; post-test, *p* < 0.01), but not PS latency as compared to HC rats given saline. In Figure 5B, the quantitative analysis showed that total sleep time in 24 h in PSD rats was increased by 21% as compared to that in HC rats (*F*_(2,22)_ = 14.730, *p* < 0.001; post-test, *p* < 0.001). Moreover, the increased PS time after PSD was due to a rise of episode number during 12-h light period and both episode number and duration during 12-h dark period (Figures 5C,D). The augment of SWS time during dark period was the result of increased episode number. The reduction of wakefulness during light and dark period was because of a significant reduction of episode duration instead of episode number (Figures 5C,D).

#### Effect of NPS on PSD-Induced Sleep-Wake Architecture

In comparison with PSD and HC rats given saline (Figures 3A,B), the PSD rats given NPS (1 nmol, Figure 3C) induced a 30-min wakefulness accompanied with fast and low-voltage activities of cortical EEG and a dense EMG activity. Subsequently, an increase of SWS accompanied with slow and high-voltage activities of EEG and a diminution of EMG activity were followed. Cortical EEG power spectral analysis for the sleep recovery showed that NPS markedly increased delta (0.5–4 Hz) activities, suppressed theta (4.5–8.5 Hz) and decreased beta and gamma (14.5–60 Hz) activities.

An analysis of hourly sleep-wake amount over 24 h revealed that PSD rats given NPS, as compared to PSD rats given saline, showed an increase in wakefulness at 8:00 h (*p* < 0.05) and during 19:00–20:00 h (*p* < 0.05) with a concomitant decrease in SWS, and a noticeably decrease in PS was during 8:00–10:00 h (*p* < 0.001, *p* < 0.01, *p* < 0.05, respectively) and 16:00–19:00 h (*p* < 0.05, *p* < 0.01, *p* < 0.01, *p* < 0.001, respectively) in light phase and at 5:00 h (*p* < 0.05) in dark phase (Figure 4A). A cumulative amount of each stage in 12-h light and dark phase, and 24-h total showed that the amount of wakefulness, SWS and PS between HC rats given saline and PSD given NPS in light phase were not significant difference. NPS decreased wakefulness and increased SWS in 12-h dark phase and 24-h total, as well as increased PS in 12-h dark phase as compared to HC rats given saline (Figure 4B). Compared to PSD rats given saline, NPS increased wakefulness by 24% in 12-h light phase (*F*_(2,22)_ = 4.512, *p* = 0.023; post-test, *p* < 0.05) and 11% in 24-h total (*F*_(2,22)_ = 11.303, *p* < 0.001; post-test, *p* < 0.05). It decreased PS by 33% in 12-h light phase (*F*_(2,22)_ = 7.803, *p* = 0.003; post-test, *p* < 0.01) and 23% in 24-h total (*F*_(2,22)_ = 14.674, *p* < 0.001; post-test, *p* < 0.01), respectively (Figure 4B).

As shown in Figure 5A, in comparison with PSD rats given saline, NPS markedly increased SWS latency (26.63 ± 3.99 min vs. 11.94 ± 2.25 min; *F*_(2,22)_ = 23.069, *p* < 0.001; post-test, *p* < 0.01) and PS latency (71.50 ± 11.64 min vs. 22.89 ± 2.71 min; *F*_(2,27)_ = 20.595, *p* < 0.001; post-test, *p* < 0.001). The quantitative analysis (Figure 5B) showed that total sleep time in 24 h in PSD rats given NPS was increased by 11% as compared to HC rats given saline (*F*_(2,22)_ = 14.730, *p* < 0.001; post-test, *p* < 0.05), but decreased by 8% as compared to PSD rats given saline (*F*_(2,22)_ = 14.730, *p* < 0.001; post-test, *p* < 0.05), suggesting that sleep amount in 24 h in PSD rats given NPS was lesser than that given saline. Moreover, compared to HC rats given saline, the increase of SWS and PS time during dark period in PSD rats given NPS was respectively due to augment of SWS episode number and of both PS episode number and duration (Figure 5D). Compared to PSD rats given saline, NPS-induced decrease in wakefulness during dark period was because of a significant reduction in episode duration (Figure 5D). NPS-induced reduction in PS time during light period was because of a decreased episode number, whereas increase in wake during light period was because of extended episode duration (Figure 5C).

### PSD Upregulated NPSR mRNA Expression Level in the Amygdala

The amygdala plays a major role in regulating anxiety (Tovote et al., 2015) and sleep-wake cycle (Sanford et al., 2002; Wellman et al., 2014). We detected NPSR mRNA expression in the amygdala of rat using RT-qPCR, and found that 24-h PSD increased the NPSR mRNA expression level by 56.6% (*t* = 4.93, *p* < 0.01; Figure 6D). The increased NPSR expression in the amygdala after PSD is presumably related to PSD-induced anxiety-like behavior and sleep disturbance.

### NPS-Induced Activated Neurons in the Amygdala Mostly Bear NPSR

#### NPS Increases the Number of Fos Immunoreactive (ir) Neurons in the Amygdala

**Figure 7:**
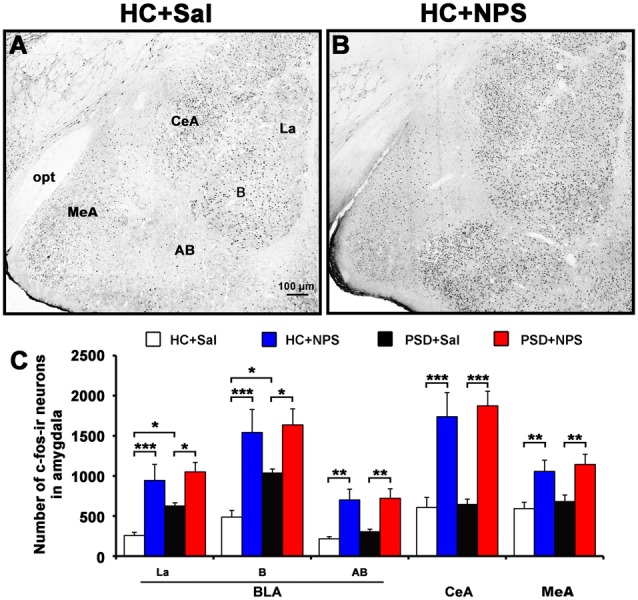
Distribution of NPS-induced Fos immunoreactive (ir) neurons in the amygdala. Microphotographs representatively show Fos-ir neurons (black dots) in the amygdala in HC rats treated with saline **(A)** and NPS **(B)**. Histograms **(C)** show quantitative analysis of the number of For-ir neurons in the basolateral amygdala (BLA) including the lateral nucleus (La), basal nucleus **(B)** and accessory basal nucleus (AB), and the central amygdala (CeA) and medial amygdala (MeA) following saline (*n* = 5) and NPS (*n* = 5) i.c.v. injection to HC and PSD rats. Values are means ± SEM. *P < 0.05, **P < 0.01, ***P < 0.001. Data were analyzed by two-way ANOVA. Bar = 100 μm.

In comparison with HC rats given saline (Figure 7A), HC rats given NPS (Figure 7B) increased the number of Fos-ir neurons by 3.3-fold (3183.8 ± 614.6 vs. 958.6 ± 142.8; *F*_(1,16)_ = 17.467, *p* < 0.001) in the BLA. When the number of cells in three areas of the BLA was counted separately, we found that NPS increased Fos-ir neurons in the lateral nucleus (La) by 3.7-fold (942.4 ± 201.4 vs. 256.8 ± 40.9; *F*_(1,16)_ = 16.340, *p* < 0.001), in the basal nucleus (B) by 3.2-fold (1540.8 ± 286.0 vs. 485.8 ± 84.2; *F*_(1,16)_ = 16.936, *p* < 0.001) and accessory basal nucleus (AB) by 3.2-fold (700.6 ± 133.2 vs. 216.0 ± 25.9; *F*_(1,16)_ = 14.088, *p* < 0.01). NPS also increased the number of Fos-ir neurons in the CeA by 2.9-fold (1737.6 ± 300.1 vs. 606.2 ± 126.4; *F*_(1,16)_ = 17.854, *p* < 0.001) and MeA by 1.8-fold (1055.6 ± 140.6 vs. 591.2 ± 80.7; *F*_(1,16)_ = 8.763, *p* < 0.01; Figure 7C). Compared to PSD rats given saline, PSD rats given NPS increased the number of Fos-ir neurons in the La by 1.7-fold (1050.2 ± 117.4 vs. 624.2 ± 39.3; *F*_(1,16)_ = 6.309, *p* < 0.05), in the B by 1.6-fold (1635.6 ± 199.9 vs. 1035.4 ± 50.7; *F*_(1,16)_ = 5.481, *p* < 0.05), in the AB by 2.4-fold (720.8 ± 118.0 vs. 303.6 ± 31.6; *F*_(1,16)_ = 10.442, *p* < 0.01), and CeA by 2.9-fold (1873.4 ± 181.4 vs. 643.4 ± 66.5; *F*_(1,16)_ = 21.102, *p* < 0.001) and MeA by 1.7-fold (1142.4 ± 127.4 vs. 680.2 ± 82.1; *F*_(1,16)_ = 8.680, *p* < 0.01). However, the amount of Fos-ir neurons between HC and PSD rats given NPS showed no significant difference. PSD rats given saline only increased the number of Fos-ir neurons in the La (*F*_(1,16)_ = 4.692, *p* < 0.05) and B (*F*_(1,16)_ = 4.596, *p* < 0.05) compared to HC rats given saline (Figure 7C).

#### NPS-Induced Activated Neuron Co-Expresses NPSR in the Amygdala

**Figure 8:**
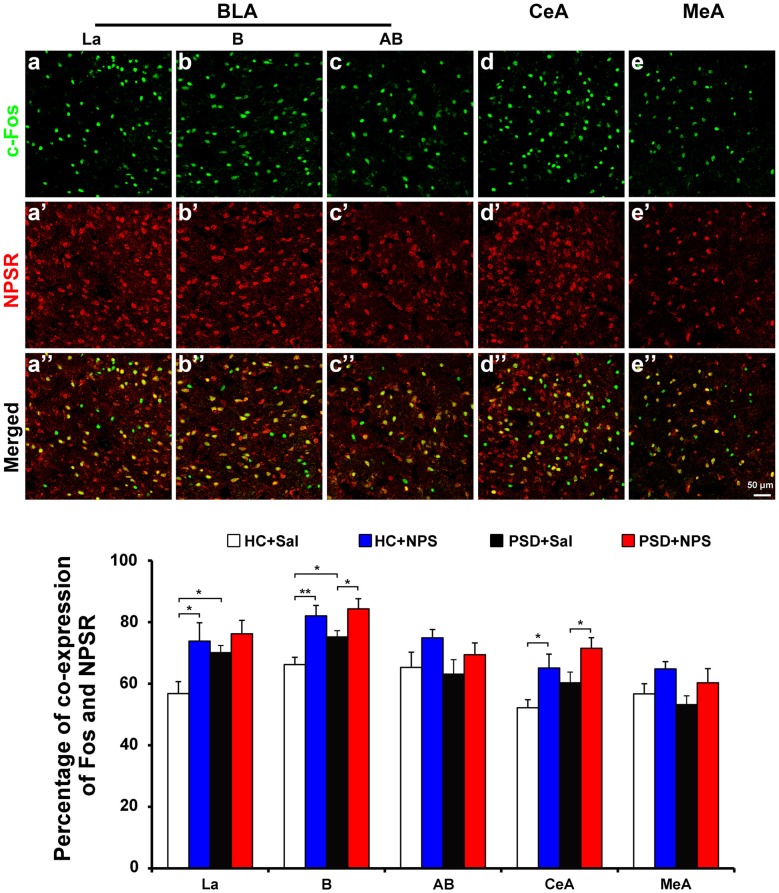
Distribution of Fos-ir neurons expressing NPSR in the amygdala. Photomicrographs representatively show Fos-ir neurons (green) in the BLA including La **(a)**, B **(b)** and AB **(c)**, the CeA **(d)** and MeA **(e)** after the HC rats given NPS, NPSR-ir neurons (red) in the La **(a’)**, B **(b’)**, AB **(c’)**, CeA **(d’)** and MeA **(e’)**, and the co-expression (yellow) of Fos-ir and NPSR-ir neurons in the La **(a”)**, B **(b”)**, AB **(c”)**, CeA **(d”)** and MeA **(e”)**, respectively. Bar = 50 μm. The histogram displays the percentage of Fos-ir neurons expressing NPSR in the amygdala (bottom panel) in HC and PSD rats given saline or NPS. Values are means ± SEM. *P < 0.05, **P < 0.01. Data were analyzed by two-way ANOVA. Abbreviations: AB, accessory basal nuclei; B, basal nuclei; BLA, basolateral amygdala; CeA, central amygdala; La, lateral nuclei; MeA, medial amygdala.

Dual-immunofluorescence for Fos and NPSR in HC rats given NPS showed that 77.9 ± 3.6% of Fos-ir neurons in the BLA were positive for NPSR. The percentage of Fos-ir neurons that also perform NPSR was 73.8 ± 6.0% in the La (Figures 8a–a″ and bottom panel), 82.0 ± 3.4% in the B (Figures 8b–b″ and bottom panel) and 74.9 ± 2.7% in the AB (Figures 8c–c″ and bottom panel), respectively. NPS-induced Fos-ir neurons that also expressed NPSR were 65.1 ± 4.5% in the CeA (Figures 8d–d″ and bottom panel) and 64.8 ± 2.4% in the MeA (Figures 8e–e″ and bottom panel). The percentage of co-expression of Fos and NPSR neurons in PSD rats given NPS was 76.2 ± 4.4% in the La, 84.3 ± 3.3% in the B and 69.4 ± 3.8% in the AB, 71.5 ± 3.5% in the CeA and 60.3 ± 4.6% in the MeA (Figure 8 bottom panel). In HC and PSD rats given saline, the percentage of co-expression of Fos and NPSR neurons respectively was 56.8 ± 3.9% and 70.1 ± 2.3% in the La, 66.2 ± 2.4% and 75.2 ± 2.0% in the B, 65.3 ± 4.9% and 63.1 ± 4.7% in the AB, 52.2 ± 2.6% and 60.3 ± 3.5% in the CeA, and 56.7 ± 3.3% and 53.2 ± 2.9% in the MeA. The comparative analysis of co-expression of Fos and NPSR neurons revealed no difference in the amygdala in HC and PSD rats given NPS, but a significant difference in the La in HC rats given saline and NPS (*F*_(1,16)_ = 7.661, *p* < 0.05) and in HC and PSD rats given saline (*F*_(1,16)_ = 4.689, *p* < 0.05). There is a significant difference in the percentage of co-expression of Fos and NPSR neurons in the B in HC and PSD rats given saline (*F*_(1,16)_ = 5.115, *p* < 0.05), in HC rats given saline and NPS (*F*_(1,16)_ = 15.765, *p* < 0.01) and in PSD rats given saline and NPS (*F*_(1,16)_ = 5.229, *p* < 0.05), respectively. The percentage of co-expression of Fos and NPSR neurons in the CeA also showed differences in HC rats given saline and NPS (*F*_(1,16)_ = 6.490, *p* < 0.05) and in PSD rats given saline and NPS (*F*_(1,16)_ = 4.892, *p* < 0.05; Figure 8 bottom panel).

## Discussion

PSD for 24 h caused anxiety-like behaviors, such as reduced central activity time and rearing number, and increased the duration of grooming in OFT, and decreased the time spent in light box and transition number between light and dark box in LDB (Figure 2). In the anxiety tests of rodent, reduction of central activity and rearing, enhancement of self-grooming, and unwillingness to explore the light and more willingness to spend more time in the dark have been considered as behavioral markers of increased anxiety-like behavior (Andersen et al., 2005; Vollert et al., 2011; Pires et al., 2013). The present study demonstrates for the first time that centrally administered NPS completely reversed the 24-h PSD-induced anxiety-like behavior (Figure 2).

Anxiety is a classic consequence of sleep deprivation in both humans and animals (Suchecki et al., 2012; Pires et al., 2013). This was first observed by Dement (1960), who reported that anxiety, in association with irritability and concentration deficits, is one of the most important neuro-behavioral consequences of PSD (Dement, 1960) and total sleep deprivation (Sagaspe et al., 2006). SWS and PS have been of significant interest to psychiatrists, because SWS plays a putative role in CNS energy recuperation and cognitive function and PS is involved in memory, mood regulation, and possible emotional adaptation (Kyung Lee and Douglass, 2010).

The rodent anxiety models are frequently employed to study the changes in sleep-wake architecture after experimental manipulation (Tang et al., 2005; Maclean and Datta, 2007). In the present study, the rats showed longer sleep latency after a 24-h PSD although they might have a stronger sleep drive for sleep recovery. The increased sleep latency is a typical characteristic of anxiety (Monti and Monti, 2000). After the long sleep latency, the rats subsequently showed a marked PS rebound accompanied by higher cortical theta band (4.5–8.5 Hz) activities across light and dark period, and an increase in SWS during dark period. The PS rebound was because of increase in the number of PS episodes (Figures 3–5). The increased number of SWS episodes lasted for 24 h, though the amount of SWS was not increased during light/inactive period. These evidences indicate that there was significant fragmentary sleep, which is a notable sign of anxiety disorder and significantly exists during sleep recovery after PSD (Jakubcakova et al., 2012). The increased PS as a result of increased number of PS episode has been considered to be a biomarker of anxiety, and is believed to be provoked by stress rather than sleep loss (Gandolfo et al., 1996). More importantly, recent studies have found that the increased theta activity of cortical and hippocampal EEG is an electrophysiological characteristic of anxiety (Yeung et al., 2012; Hoeller et al., 2013). PS is well known to play a fundamental role on the emotional and mental recovery from adverse situations. It has been proposed that one of the functions of PS is to weaken undesirable and persistent memories (Crick and Mitchison, 1983; Suchecki et al., 2012). Moreover, sleep deprivation-induced anxiety is a very labile behavior, which, regardless of the severity, is easily reversed by the restoration of normal sleep patterns (Dement, 1960). Thus, PS rebound is not only altered by stress but also by inadequate sleep in PSD animals (Suchecki et al., 2002). These results of our study give valuable insights into how the sleep-wake pattern affects anxiety-like behavior induced by PSD.

Central administration of NPS not only improved PSD-caused anxiety-like behavior in rats, but also altered PSD-induced sleep-wake pattern and cortical EEG power spectrum. In the present study, NPS (1 nmol) induced wakefulness for 41 min in the 1st hour after 24-h PSD in rats. It was reported that NPS administration (1 nmol, i.c.v.) enhanced wakefulness for 3 h in normal rats (Zhao et al., 2012). The decreased arousal effect of NPS might be due to PSD-induced sleep drive. In addition, the amount of wakefulness, SWS and PS between PSD rats given NPS and home cage control rats in light phase were not significantly different (Figure 4B), suggesting that NPS might ameliorate PSD-induced sleep disturbance. It is worth noting that NPS persistently suppresses PS for 3 h in both PSD and normal rats (Zhao et al., 2012). The mechanisms underlying NPS-induced PS suppression are yet to be elucidated. Several studies have demonstrated that increased PS episode number and cortical EEG of theta activity are characteristics of anxiety-like sleep, whereas suppression of theta activities of hippocampus is considered to be a biomarker of anxiolytics (Gandolfo et al., 1996; Yeung et al., 2012; Hoeller et al., 2013). These features of suppression of PS and cortical theta activities by NPS in current study provide insights into the mechanisms how NPS alters sleep pattern.

The anxiolysis and arousal effect of NPS-NPSR system have been identified by several studies (Xu et al., 2004; Jüngling et al., 2008; Enquist et al., 2012; Zhao et al., 2012). This unique behavioral profile challenges the conventional theory that anxiolytics are also sedative (i.e., benzodiazepines) and stimulants are also anxiogenic (i.e., caffeine, cocaine and amphetamines). Nicotine shares this behavioral profile with NPS, and it increases arousal and produces, at least in smokers, anxiolysis and anti-stress effects (Guerrini et al., 2010). Interestingly, nicotine is also thought to act via regulation of the endogenous NPS-NPSR system because it increases NPS expression in the brainstem and NPSR expression in both brainstem and hypothalamus in rats (Lage et al., 2007). These evidences, together with our findings that NPS reduced PSD-induced sleep rebound (Figure 5B), suggest that NPS and other agonist of NPSR may become effective anxiolytics which do not cause subsequent sleep rebound or hypersomnia.

To further clarify the mechanism of anxiety-related behavior induced by 24-h PSD and identify the potential therapeutic targets, we determined the dynamic alteration of NPSR mRNA in the amygdala which is well known to play the key role in mediating anxiety and anxiolysis (Grupe and Nitschke, 2013; Tovote et al., 2015). We found that PSD significantly increased the level of NPSR mRNA expression (Figure 6). The findings that PSD induced anxiety-like behaviors and enhanced NPSR expression in rats, and that NPS improved anxiety indicate that the NPSR is a reasonable target for the intervention in PSD-induced anxiety-like behavior. There were also reports that NPS failed to show anxiolytic effect in NPSR-knockout mice (Duangdao et al., 2009; Zhu et al., 2010). In animals with alcohol withdrawal-induced anxiety, NPSR gene expression was also increased in the MeA, BLA and CeA (Ruggeri et al., 2010). The increase in NPSR gene expression in the amygdala has been considered to be a compensatory mechanism to reduce anxiety occurring in animals with a history of dependence (Ghazal et al., 2013). In addition, several studies in humans demonstrated that the functional polymorphism Asn^107^Ile (rs324981, A > T) of the NPSR gene is associated with anxiety disorders (Domschke et al., 2011; Neufang et al., 2015). Collectively, the findings demonstrated that the upregulation of the NPSR transcript leads to an increase of the NPS function, which supports the hypothesis that increased expression of brain NPSR is a part of compensatory neuroadaptive changes and homeostatic regulation aimed at reducing anxiety associated with PSD. These findings also suggest that NPS-NPSR system is presumably involved in the regulation of anxiety and anxiolysis.

To identify the target cells through which NPS facilitates anxiolytic effect, we labeled activated neurons by staining of c-Fos, the product of the immediate early gene that is expressed in association with neuronal activation (Zhao et al., 2012; Shao et al., 2013, 2016). We also did double staining of c-Fos and NPSR in the amygdala to determine if NPS activated neurons are ones that express NPSR. The results show that both HC and PSD rats given NPS significantly increased the number of Fos-ir neurons in the BLA, CeA and MeA (Figure 7), and most of the activated cells also expressed NPSR (Figure 8). Interestingly, PSD increased the number of Fos-ir neurons in the La and B of BLA as compared to HC rats given saline (Figure 7). Because BLA mediates both anxiogenic and anxiolytic behavioral effects, there must be distinct neuronal circuits in anxiety. Several previous studies have demonstrated that the somatic activation of BLA projection neurons results in enhanced anxiety-like behavior, while the selective activation of excitatory BLA axonal projections that terminate in the CeA is anxiolytic (Tye et al., 2011; Tovote et al., 2015). NPS injected into the BLA or CeA promotes anxiolysis (Fendt et al., 2010; Chauveau et al., 2012; Enquist et al., 2012), and emotional stresses induce release of NPS in the amygdala (Ebner et al., 2011). An increasing number of studies have shown that MeA is also involved in generalized anxiety disorder (Herdade et al., 2006). Central administration of NPS enhances GABAergic activities in MeA interneurons, resulting in the increased intra-amygdaloidal inhibitory transmission to alleviate nociception-induced anxiety-like behaviors (Zhang et al., 2014). In addition, the amygdala is also involved in mediating the effects of emotions/stress on sleep. For example, the amygdala, through its noradrenergic afferents, contributes to the sleep rebound mechanisms after sleep deprivation (Charifi et al., 2000), whereas inactivating CeA can produce a relatively selective suppression of PS (Sanford et al., 2002). The BLA is an important regulator of stress-induced alterations in sleep (Wellman et al., 2014). In other words, the CeA is more involved in the regulation of PS while the BLA has a greater role in the regulation of SWS sleep and arousal. However, it is important to note that BLA regulates CeA output and therefore likely controls its influences on PS (Sanford et al., 2015). In our previous studies, we found that NPS i.c.v. injection also induced an increase in the number of Fos-ir neurons in posterior hypothalamic, subiculum complex, olfactory cortex, perifornical nucleus and other nuclei, so the present study could not exclude the possibility that other neuronal circuits may be involved in the regulation of anxiety and sleep too. Nonetheless, our data indicate that NPS counteracts PSD-induced anxiety-like behavior, and alters PSD-induced sleep disturbances probably through activation of neurons bearing NPSR in the amygdala.

## Conclusion

The central action of NPS counteracts PSD-induced anxiety-like behavior, and alters PSD-induced sleep disturbances by characteristically increasing wakefulness, and suppressing PS and cortical theta activity. PSD enhances NPSR mRNA expression level in the amygdala. NPS markedly increases the number of Fos-ir neurons in the BLA, CeA and MeA. The majority of Fos-ir neurons induced by NPS also express NPSR. Upregulation of the NPSR transcript is likely a compensatory neuroadaptive change and homeostatic regulation aimed at increasing NPS function to reduce PSD-caused anxiety. NPS counteracts PSD-induced anxiety-like behavior and sleep disturbances probably through activation of the NPSR in the neurons of the amygdala. The fact that PSD rats treated with NPS showed little sleep increase suggests that NPS or other effective agonist of NPSR may become an activate anxiolytic which does not cause sleep rebound or hypersomnia.

## Author Contributions

Y-PH, J-FX and Y-FS designed the study. J-FX, H-LW, CW and L-XW conducted the experiments. X-PK, G-FC, Y-NC, C-YC and H-LC acquired the data. J-FX, H-LW, Y-FS and Y-PH analyzed the data. Y-PH, J-FX and Y-FS drafted the manuscript, which all other authors revised. All authors approved the final version and evaluated the accuracy and integrity of the work.

## Conflict of Interest Statement

The authors declare that the research was conducted in the absence of any commercial or financial relationships that could be construed as a potential conflict of interest.
